# RiSID: River Surface Image Dataset for Instance Segmentation of Floating Macroplastic Debris

**DOI:** 10.1016/j.dib.2025.112189

**Published:** 2025-10-16

**Authors:** Tomoya Kataoka, Takushi Yoshida, Natsuki Yamamoto

**Affiliations:** a Department of Civil & Environmental Engineering, Ehime University, 3 Bunkyo-cho, Matsuyama, 790-8577, Japan; b Center for Marine Environmental Studies, Ehime University, 3 Bunkyo-cho, Matsuyama, 790-8577, Japan; c Business Planning and Development Division, Yachiyo Engineering Co. Ltd., 5-20-8, Asakusabashi, Taito-ku, Tokyo, 111-8648, Japan

**Keywords:** Floating macroplastic debris, Instance segmentation, River surface, MS COCO

## Abstract

Rivers constitute a major pathway of macroplastic debris, which has the potential to have adverse impacts on marine ecosystems and oceans. It is essential to develop image-based technology for quantifying macroplastic debris floating on river surfaces and then grasping plastic transport from land. The river surface image dataset (RiSID) comprises 7,356 original images recorded at 11 sites on seven rivers during high-flow conditions in Japan, along with pixelwise segmentation annotations for floating macroplastic debris. The three annotation datasets were divided into seven, five, and two categories of floating anthropogenic debris to explore the model performance. The annotation data were packaged in a JSON file in the Microsoft Common Objects in Context (MS COCO) format, which is a common format for computer vision research on developing deep learning models. RiSID would be helpful for researchers to explore a better model for monitoring floating macroplastic debris via river surface images.

Specifications Table

**Table:** 

Subject	Earth & Environmental Sciences
Specific subject area	*Detection/classification of floating macro plastic debris on river surface via a deep learning approach with instance segmentation architecture.*
Type of data	*Image, JSON, Processed*.
Data collection	*Video footage was captured at 11 sites on seven Japanese rivers using cameras either fixed to bridge rails or held by hand, with the river surface recorded in a perpendicular downward view from the bridges. From the collected footage, 7,356 frames containing visible target objects were selected. A total of 8,022 target objects were annotated using EISeg, an open-source interactive segmentation tool, and categorized into seven, five, or two classes. The annotation data were stored in JSON files following the MS COCO format, including class labels and segmentation masks, which can be used for training and evaluating instance segmentation models.*
Data source location	*The image data were collected at 11 sites on the seven Japanese rivers, which are listed in*Table 2*.*
Data accessibility	Repository name: zenodo Data identification number: 10.5281/zenodo.16927238 Direct URL to data: https://doi.org/10.5281/zenodo.16927238
Related research article	*T. Kataoka, T. Yoshida, N. Yamamoto, Instance segmentation models for detecting floating macroplastic debris from river surface images, Frontiers in Earth Science 12 (2024),*https://doi.org/10.3389/feart.2024.1427132*.*

## 1 Value of the Data

• The river surface image dataset (RiSID) comprises 7,356 image files in PNG format, and the JSON files store 8,022 annotation data of macroplastic debris on the river surface.
• The image data were collected at 11 sites in seven Japanese rivers under high-flow conditions, in which plastic objects flow down with various natural materials, such as vegetation.
• The JSON files contain the pixelwise segmentation annotations of floating macroplastic debris observed on river surfaces, which can be used for developing a deep learning model with an instance segmentation architecture for plastic detection.
• We prepared three annotation datasets in which common plastic objects in river environments were grouped into seven, five, or two categories to investigate how the performance of the deep learning model depends on category selection.
• The annotation data follow the Microsoft Common Objects in Context (MS COCO) format for sharing and include class labels and segmentation masks, which is a common format in computer vision research for developing deep learning models.
• RiSID can be used to benchmark segmentation models (e.g., Mask R-CNN and YOLO segmentation) for detecting and classifying floating macroplastic debris, thereby contributing to the acceleration of model development within the research community.

## 2 Background

Most of the ubiquitous macroplastic debris in aquatic environments is emitted from land via rivers, which are considered major pathways to the ocean [1]. In particular, quantifying macroplastic debris floating on river surfaces is crucial for understanding plastic emissions from land. Thus, a number of researchers have attempted to detect/classify macroplastic debris via a deep learning approach [2]. The instance segmentation architecture can generate pixelwise segmentation as well as object detection, as the pixelwise segmentation may serve as a useful approach for reducing uncertainty in the evaluation of mass transport of floating macroplastic debris [3]. Hence, the application of instance segmentation enables the evaluation of plastic fluxes in terms of quantity and mass. The bottleneck to develop the plastic-detection model is to create the dataset annotated floating macroplastic debris because it is time-consuming and hard work.

Here, we share the annotation data in the development of the instance segmentation model for detecting/classifying floating macroplastic debris. Our dataset is expected to accelerate the development of instance segmentation models and contribute to the evaluation of their performance.

## 3 Data Description

**Fig. 1:**
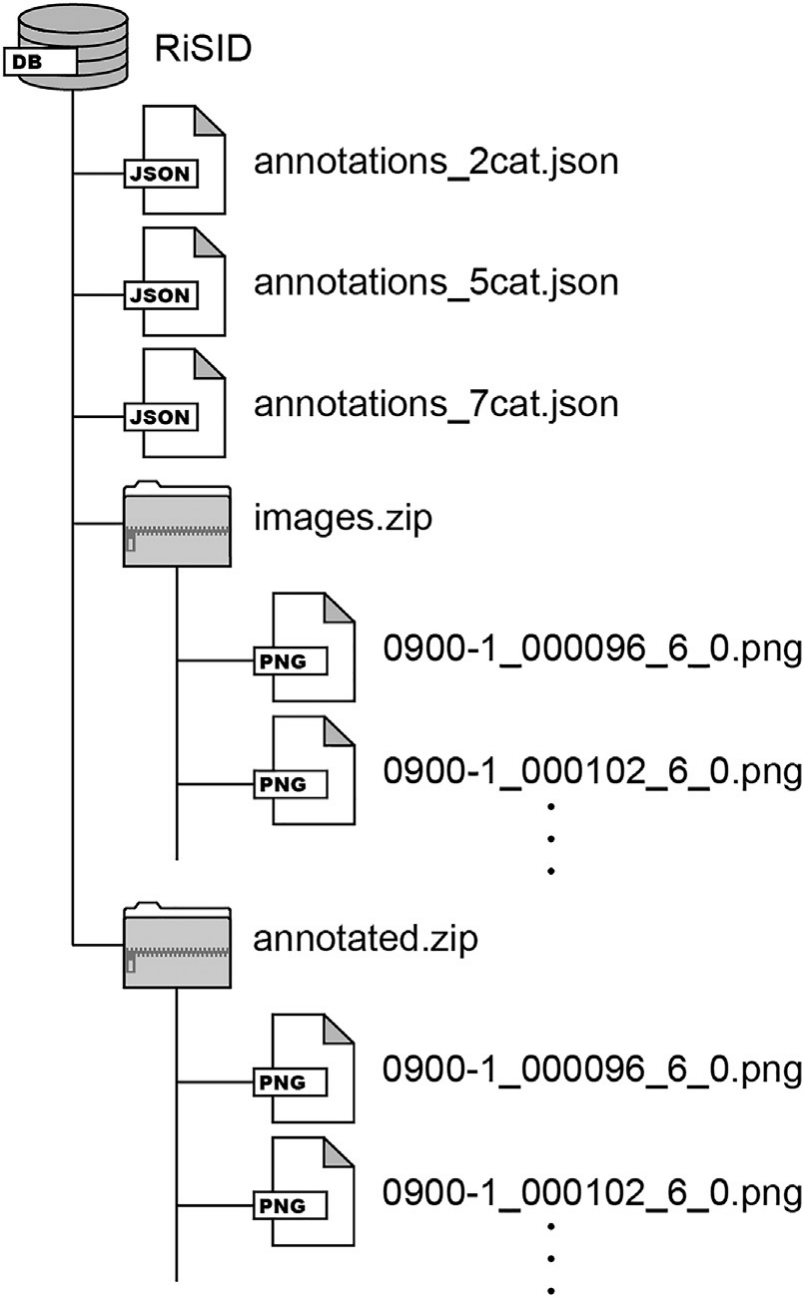
River surface image dataset (RiSID) includes three JSON files for annotations and two ZIP files containing original and annotated image files, all in PNG format.

The dataset consists of images and corresponding JSON files (Fig. 1). All original images are stored in PNG format, which are provided within the “images” directory. To visualize the annotation data, all images annotated with the seven-class labels are provided within the “annotated” directory. Three types of JSON files with different numbers of classes are included. This design aims to investigate the dependency of deep learning model performance on the number of object classes. Details of these images and their corresponding JSON files are described below.

### 3.1 Original image dataset

**Fig. 2:**
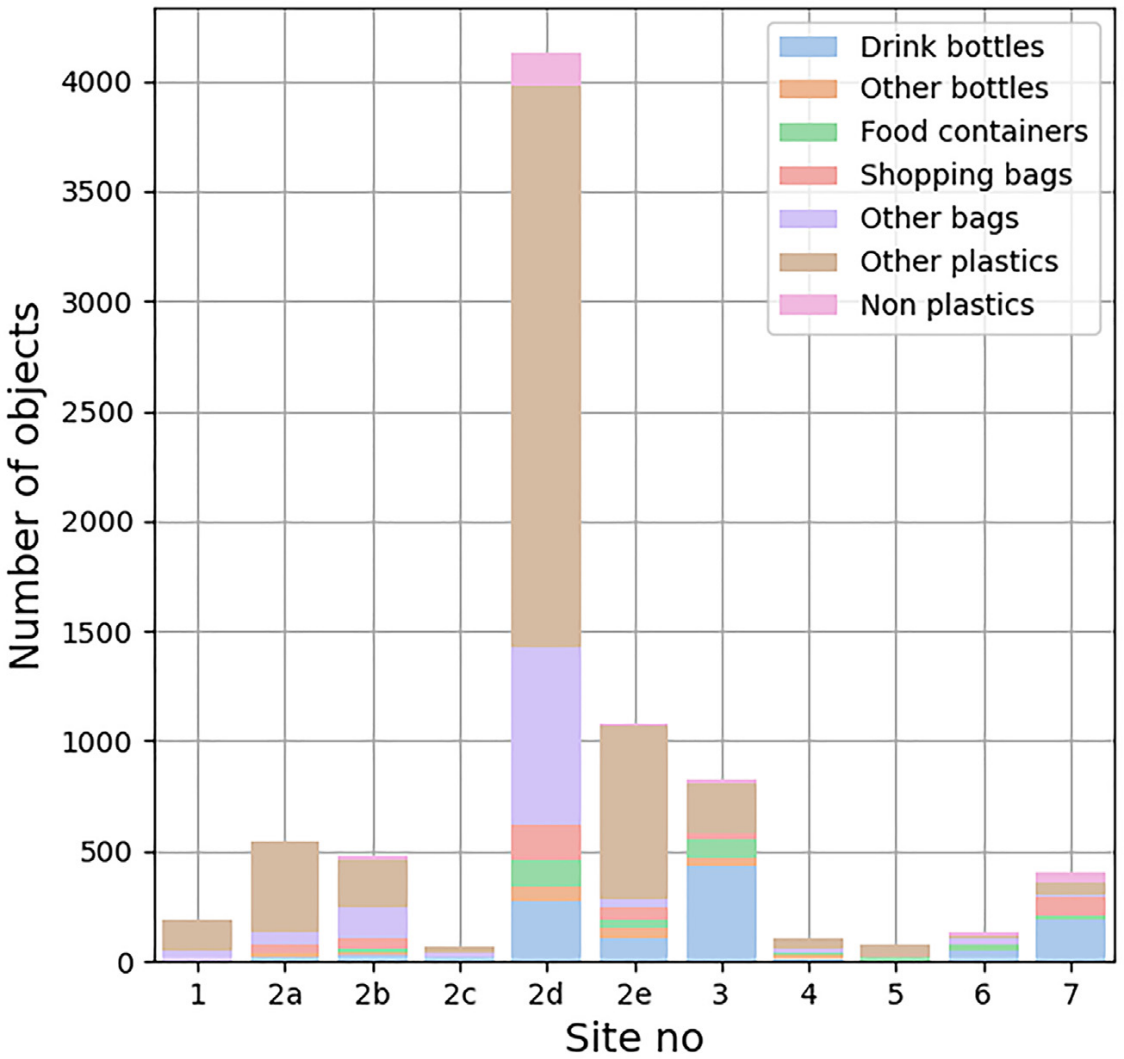
The number of annotated objects at each site is shown. The site numbers correspond to Table 2.

The dataset of floating riverine plastic debris contains 7,356 river surface images. Most of the images contain a single target object, although multiple objects are occasionally present. In total, 8,022 objects were found in all the images (Fig 2). Their sizes are standardized to 1,024 px × 1,024 px to improve the efficiency of training and inference processes by the trained deep learning model [3]. And the RiSID shows a significant imbalance in the number of objects per category, denoting the tendency of floating riverine plastic debris in Japanese rivers. To improve robustness in model development, users may need to consider any techniques like, weighted loss functions [4], data augmentation [5].

### 3.2 JSON dataset

The 8,022 objects were divided into seven, five or two classes. In the seven-class JSON file, all objects were categorized as “drink bottles”, “other bottles”, “food containers”, “shopping bags”, “other bags”, “other plastics”, or “nonplastics” (Table 1). The five classes in the JSON file were categorized as “drink bottles”, “food containers”, “shopping bags”, “other plastics”, and “nonplastics”. The two-class JSON files were categorized as “plastic” or “nonplastic”. Notably, the classes “other bottles”, “other bags”, and “other plastics” were merged into one class, “other plastics”, for the five-class pattern, and then the six classes except “nonplastics” were merged into one class, “plastics”, for the two-class pattern. The class “nonplastic” contains other anthropogenic debris, such as aluminum/steel cans.

**Table 1:** Examples of annotation data of each category

The accompanying JSON file follows the Microsoft Common Objects in Context (MS COCO) format and comprises four primary sections: “info”, “categories”, “images”, and “annotations”. The “info” section contains metadata describing the overall dataset. The “categories” section contains information about each object class, including a unique class ID (“id”), class name (“name”), associated color (“color”), and the supercategory name (“supercategory”). The “id” field is uniquely assigned to each class and is referenced in the “annotations” section as “category_id”. In the seven-class pattern, the “name” field includes seven categories: “bottle” (drink bottles), “o_bottle” (other bottles), “food_pack” (food containers), “bags” (shopping bags), “o_bag” (other bags), “o_pla” (other plastics), and “non_pla” (nonplastics). In the five-class pattern, the “name” field includes five categories other than “o_bottle” and “o_bag”. In the two-class pattern, the “name” field includes just two categories: “pla” (plastics) and “non_pla” (nonplastics). The “supercategory” field is set to null for all entries in this dataset. “”

The “images” section lists all the images included in the dataset, with each entry containing a unique ID (“id”), file name (“name”), and image dimensions (“height” and “width”). The “id” field is referenced in the “annotations” section as “image_id”. In this dataset, the keys “coco_url”, “date_captured”, and “flickr_url” are assigned null values.

The “annotations” section includes the segmentation mask (“segmentation”), area (“area”), the ID of the corresponding image (“image_id”), bounding box (“bbox”), class ID of the object (“category_id”), and a unique annotation ID (“id”) assigned to each object. The keys “num_keypoints”, “keypoints”, “iscrowd”, and “caption” are set to null in this dataset.

## 4 Experimental Design, Materials and Methods

**Fig. 3:**
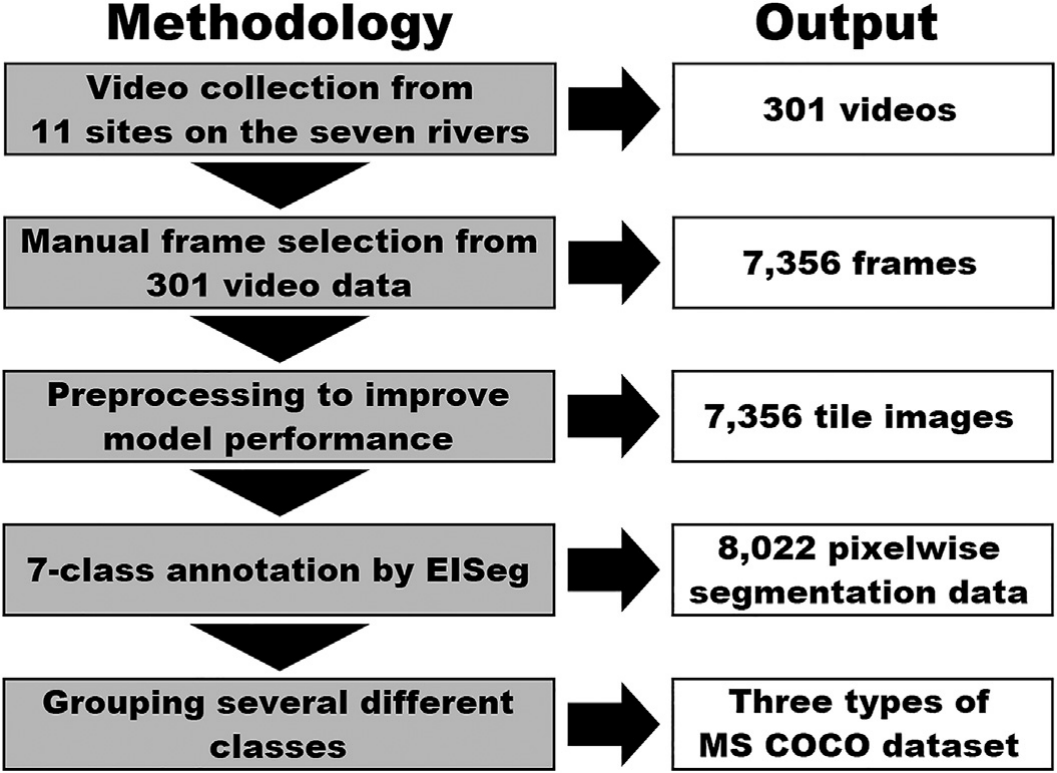
Step-by-step workflow for creating RiSID.

RiSID was developed following the step‑by‑step workflow shown in Figure 3. Each step is described below.

The 301 video data of river/waterway surfaces viewed perpendicularly downward from bridges were collected at 11 sites on the seven rivers by video cameras fixed on a bridge rail or held by hand (Table 2). The specifications of the video cameras used in this study varied. The recorded videos consisted of two image resolutions—HD and full HD—with frame rates ranging from 10–30 frames per second. All videos were encoded via the H.264 compression format with the MPEG-4 codec. The recording periods also varied, but the dataset primarily comprises videos captured during flood seasons. This diversity in recording conditions was intentional, aiming to enhance the generalization performance of instance segmentation models developed and evaluated using the dataset.

**Table 2:** Quantity of data, camera specifications and monitoring periods at each site

	**River/Channel**	**Site**	**Coordinates**	**Collected movies**	**Selected images**	**Image Size**	**Focal length [mm]**	**Senor size [inch]**	**Framerate [fps]**	**Installation periods**
1	Arakawa R.	Nishi-arai	35°45’31.51’’N 139°47’21.51’’E	2	163	HD (1280×720)	4.4-88	1/2.5	29.97	Feb. 16, 2021
2a	Danzu R.	Jindo	34°44’19.9’’N 137°43’5.00’’E	41	531	HD (1280×720)	2.8	1/2.7	10	Sep. 9, 2022-Jun. 29, 2023, Mar. 5-Jan. 11, 2024
2b	Danzu R.	Kamidanzu	34°44’57.64’’N 137°43’27.10’’E	31	472	HD (1280×720)	2.8	1/2.7	10	Sep. 9, 2022-Apr. 14, 2023
2c	Danzu R.	Shintomitsuka	34°43’18.62’’N 137°43’3.10’’E	3	45	Full HD (1920×1080)	2.8	1/2.7	30	Oct. 15, 2021-Jan. 14, 2022
2d	Danzu R.	Taisho	34°43’56.11’’N 137°42’50.84’’E	97	3763	HD (1280×720)	2.8	1/2.7	10	Sep. 27, 2022-Jun. 29, 2023, Dec. 6, 2023-Mar. 5, 2024
2e	Danzu R.	Usagi	34°43’33.87’’N 137°42’25.37’’E	50	1038	HD (1280×720)	2.8	1/2.7	10	Sep. 9, 2022-Apr. 14, 2023
3	Edo R.	Noda	35°56’22.80’’N 139°50’51.20’’E	44	723	Full HD (1920×1080)	29.8-298	1/2.88	29.97	Sep. 24-25, 2010, Oct. 29-31, 2010
4	Hikiji R.	Ishikawa	35°22’20.48’’N 139°27’17.18’’E	5	104	Full HD (1920×1080)	41.1-706	1/6	30	Oct. 10, 2020
5	Kamoe Ch.	Hikoo	34°42’0.00’’N 137°42’0.10’’E	4	49	Full HD (1920×1080)	2.8	1/2.7	30	Oct. 15, 2021-Jan. 14, 2022
6	Nakasuka Ch.	Nakasuka	33°51’51.73’’N 132°43’11.87’’E	11	131	Full HD (1920×1080)	2.7-13.5	1/2.5	25	Jun. 10-Oct. 27, 2022
7	Yokkaichi Ch.	Hinaga	34°56’47.26’’N 136°36’41.74’’E	13	337	HD (1280×720)	2.7-13.5	1/2.9	15	Aug. 21, 2019-Mar. 30, 2021
Total				301	7356					

From those video data, 7,356 frames in which target objects were visible were selected from numerous frames divided across all the video data. During this process, an annotator manually selected image frames in which plastic debris was clearly visible. Preprocessing was subsequently used to improve the performance and efficiency of the model [6]. The size of each image was unified by cropping the original frame to several tile images with 1,024 px × 1,024 px. When cropping the 1,024 × 1,024 px tile images, the vertical and horizontal positions of the target objects were determined using random values. This approach was adopted to avoid spatial bias during model development by preventing the debris from being concentrated in specific regions within the tile images.

The annotation data for detecting floating macroplastic debris were compiled via the efficient interactive segmentation tool (EISeg) [7]. EISeg is an open-source software that can accurately and efficiently generate segmentation masks. The target objects found from all the frames were categorized into seven debris types that are common in the seven rivers: “drink bottles”, “other bottles”, “food containers”, “shopping bags”, “other bags”, “other plastics”, and “nonplastics”. The annotation process was carried out by at least two annotators based on the following guidelines. First, to ensure consistency in the seven-category classification, a category list (as shown in Table 1) was prepared. One annotator conducted the annotation according to this list. When encountering objects that were difficult to classify, the annotators discussed the cases and, if necessary, revised the category list to include new entries. To ensure objectivity, the annotation results were subsequently reviewed by another annotator, providing a double-check of the labels.

Furthermore, to explore the dependence of the detection/classification performance on category selection, two datasets were prepared: five-class patterns and two-class patterns. The classes of “other bottles” and “other bags” were replaced with the class of “other plastics” in the five-class pattern, and then the six classes except “nonplastics” were replaced with the class of “plastics” in the two-category pattern. The annotation data follow the MS COCO format for sharing and include class labels and segmentation masks, which can be used for training and evaluating instance segmentation models.

## Limitations

The RiSID dataset offers valuable annotated data for the development of instance segmentation models to detect and clarify floating macroplastic debris, several limitations should be considered when applying the RiSID dataset to new modeling tasks or geographic contexts as follows.

Firstly, the image data was collected exclusively from Japanese rivers. As such, the types of debris, river surface conditions, and background features may differ from those in other geographic regions, which may affect the generalizability of trained models to global riverine environments. Most of these corrected images were captured during high-flow conditions, when macroplastic debris is more visible. Consequently, the dataset may not fully represent river surface appearances under low-flow or calm conditions. In such conditions, models trained on RiSID may exhibit an increased false positive rate due to misclassification of water reflections, shadows, or vegetation.

Although the dataset includes seven predefined debris categories, the annotated plastic items are limited to those commonly observed in the selected rivers. Less frequent or region-specific items may not be well represented. Moreover, annotations were generated by at least two annotators for double-checking. While this approach provides a balance between accuracy and labor efficiency, some degree of subjectivity or labeling inconsistency may remain, especially for small or partially submerged objects. If a model trained using the RiSID frequently produces false positives, several countermeasures may need to be considered. For example, it is recommended to exclude small objects based on the “area” key values in the “annotations” section of the JSON file, or to remove ambiguous images, such as those containing partially submerged objects.

## Ethics Statement

The authors have read and follow the ethical requirements for publication in Data in Brief and confirming that the current work does not involve human subjects, animal experiments, or any data collected from social media platforms.

## Credit Author Statement

**Tomoya Kataoka**: Conceptualization, Methodology, Validation, Data Curation, Writing – Original Draft, Visualization, Supervision, Project administration, Funding acquisition. **Takushi Yoshida**: Methodology, Validation, Investigation, Resources, Data Curation. **Natsuki Yamamoto**: Methodology, Validation, Investigation, Resources, Data Curation, Writing – Review & Editing.

## Data Availability

zenodoRiSID: River Surface Image Dataset for Instance Segmentation of Floating Macroplastic Debris (Original data)

## Appendix Supplementary materials

Supplementary Text S1: “Datasheet for Datasets” is provided as a supplementary text to align with the best practices for transparency and responsible data in the machine learning community [*8*]. Image, application 1

Supplementary Table S1: A manifest file (CSV format) that maps each of the 7,356 images to their source video among the 301 collected videos. This file allows researchers to trace the origin of each image and analyze potential sampling biases related to cameras or collection periods without accessing the raw video data. Image, application 2
