# Supplementary material for: RiSID: River Surface Image Dataset for Instance Segmentation of Floating Macroplastic Debris
Source: Data Brief. 2025 Oct 16;63:112189. doi: 10.1016/j.dib.2025.112189 (PMC12594929; doi:10.1016/j.dib.2025.112189)
Supplement: Supplementary file 1 — Supplementary Text S1: “Datasheet for Datasets” is provided as a supplementary text to align with the best practices for transparency and responsible data in the machine learning community [8]. [file mmc1.docx]

Supplementary Materials

RiSID: River Surface Image Dataset for Instance Segmentation of Floating Macroplastic Debris

Tomoya Kataoka^a,b^, Takushi Yoshida^c^, Natsuki Yamamoto^c^

^a^ Department of Civil & Environmental Engineering, Ehime University, Matsuyama, Japan

^b^ Center for Marine Environmental Studies, Ehime University, Matsuyama, Japan

^c^ Business Planning and Development Division, Yachiyo Engineering Co., Ltd., Tokyo, Japan

**Corresponding author:**

* Tomoya Kataoka, Department of Civil & Environmental Engineering, Ehime University, 3 Bunkyo-cho, Matsuyama, Japan, 790-8577; Tel.: +81-89-927-9817; emails: [kataoka.tomoya.ab@ehime-u.ac.jp](mailto:kataoka.tomoya.ab@ehime-u.ac.jp), [tkata@cee.ehime-u.ac.jp](mailto:tkata@cee.ehime-u.ac.jp)

Supplementary Text S1: Datasheet for Datasets

1. **MOTIVATION**

- Purpose: The dataset was created to advance research on floating macroplastic monitoring by providing annotated river surface images for instance segmentation tasks.
- Intended Use: Primarily for training and evaluating deep learning models in instance segmentation of macroplastic debris in river environments.
- Motivation: The emission of floating macroplastic debris from rivers substantially contributes to the accumulation of marine litter; however, quantitative monitoring methods for such debris remain limited. This dataset addresses this gap by providing high-quality annotations of floating macroplastic debris.

1. **COMPOSITION**

- Instances: 7,356 video frames were selected from recordings at 11 sites across seven Japanese rivers.
- Annotations: 8,022 target objects were annotated using the open-source tool EISeg.
- Categories: Three types of annotation datasets are provided: 7-class labels (“drink bottles”, “other bottles”, “food containers”, “shopping bags”, “other bags”, “other plastics”, or “nonplastics”), 5-class labels (“drink bottles”, “food containers”, “shopping bags”, “other plastics”, and “nonplastics”), 2-class labels (“plastic”, and “nonplastics”).
- Data Type: Images (PNG format) with corresponding segmentation masks in MS COCO JSON format.
- Sensitive Data: No personally identifiable information is included.

1. **COLLECTION PROCESS**

- Collection Method: 301 videos were captured using video cameras, either fixed on bridge rails or handheld, with the lens pointing perpendicularly downward to the river surface.
- Locations: Seven rivers in Japan, recorded at 11 bridge sites.
- Time Frame: Specific survey dates are provided in the main article (see Table 2).
- Selection Criteria: Frames containing visible target objects (floating macroplastics or other debris) were extracted from raw video footage.

1. **PREPROCESSING/CLEANING/LABELING**

- Preprocessing: Frames containing floating macroplastic debris were extracted from continuous video recordings.
- Labeling: Instance segmentation was performed using EISeg, supported by expert supervision to reduce subjectivity.
- Quality Control: Ambiguities were minimized by annotator training and expert cross-checking, although small or partially submerged objects remain challenging

1. **USES**

- Intended Uses: Development and benchmarking of deep learning models for debris detection and segmentation; Comparative studies of model performance under different category granularity (7, 5, 2 classes).
- Out-of-scope Uses: Not suitable for individual identification, surveillance, or any use involving personal data. Not designed for fine-scale hydrodynamic studies (e.g., flow field estimation).

1. **DISTRIBUTION**

- License: CC BY 4.0 (Creative Commons Attribution 4.0 International).
- Access: Available via the Zenodo repository (https://doi.org/10.5281/zenodo.15533743).
- Format: ZIP files containing PNG image data (original or annotated), JSON annotation files following the MS COCO format and python script to quickly start.
- Versioning: Current release is version 1 (RiSIDv1). Updates, if any, will be released under incremented version numbers

1. **MAINENANCE**

- Maintainers: The dataset is maintained by Tomoya Kataoka and colleagues.
- Update Policy: At present, no regular updates are planned, but future expansions (e.g., new sites or seasons) may be added as separate versions.
- Contact: Corresponding author’s institutional email address (listed in the article)
